# Comparative mitochondrial genomes of four species of *Sinopodisma* and phylogenetic implications (Orthoptera, Melanoplinae)

**DOI:** 10.3897/zookeys.969.49278

**Published:** 2020-09-17

**Authors:** Qiu Zhongying, Chang Huihui, Yuan Hao, Huang Yuan, Lu Huimeng, Li Xia, Gou Xingchun

**Affiliations:** 1 Shaanxi Key Laboratory of Brain Disorders &School of Basic Medical Sciences, Xi’an Medical University, Xi’an,710021, China Xi’an Medical University Xi’an China; 2 College of Life Sciences, Shaanxi Normal University, Xi’an 710062, China Shaanxi Normal University Xi’an China; 3 Key Laboratory for Space Bioscience & Biotechnology, School of Life Sciences, Northwestern Polytechnical University, Xi’an 710072, China Northwestern Polytechnical University Xi’an China; 4 Huizhou No.8 High School, Hui’zhou 516001, China Huizhou No.8 High School Hui’zhou China

**Keywords:** mitogenome, phylogeny, *
Sinopodisma
*

## Abstract

In this study, the whole mitochondrial genomes (mitogenomes) from four species were sequenced. The complete mitochondrial genomes of *Sinopodisma pieli*, *S. houshana*, *S. qinlingensis*, and *S. wulingshanensis* are 15,857 bp, 15,818 bp, 15,843 bp, and 15,872 bp in size, respectively. The 13 protein-coding genes (PCGs) begin with typical ATN codons, except for COXI in *S. qinlingensis*, which begins with ACC. The highest A+T content in all the sequenced orthopteran mitogenomes is 76.8% (*S. qinlingensis*), followed by 76.5% (*S. wulingshanensis*), 76.4% (*S. pieli*) and 76.4% (*S. houshana*) (measured on the major strand). The long polythymine stretches (T-stretch) in the A+T-rich region of the four species are not adjacent to the trnI locus but are inside the stem-loop sequences on the major strand. Moreover, several repeated elements are found in the A+T-rich region of the four species. Phylogenetic analysis based on 53 mitochondrial genomes using Bayesian Inference (BI) and Maximum Likelihood (ML) revealed that Melanoplinae (Podismini) was a monophyletic group; however, the monophyly of *Sinopodisma* was not supported. These data will provide important information for a better understanding of the phylogenetic relationship of Melanoplinae.

## Introduction

The insect mitochondrial genome (mitogenome) is a circular double-stranded covalently closed DNA molecule, with maternal genetic characteristics of relatively small molecular mass, simple structure, high copy number, relatively conservative gene arrangement, and rapid rate of gene evolution. The mitogenome contains 13 protein-coding genes (PCGs), two ribosomal RNA genes (rRNAs), 22 transfer RNA genes (tRNAs), and one A+T-rich region. The mitochondrial genes have been widely used in identifying species, estimating evolutionary relationships and recognising both the population structure and phylogeography (Flook and Rowell 1997a; Liu et al. 2013; Cameron 2014; Song et al. 2015; Du et al. 2017; Li et al. 2017; Sun et al. 2017; Zhou et al. 2017; Tang et al. 2018; Zhang et al. 2018; Chang et al. 2020). With the advancement of high-throughput sequencing technology, more and more mitogenome sequences have been sequenced. Many species systems, especially insect phylogenetic relationships, were constructed through the complete sequences of mitogenomes, complementing morphological classification (Gen 2015; Song et al. 2015; Li et al. 2017; Zhou et al. 2017; Li et al, 2020). Comparison of mitogenomes may reveal important genome-level characteristics, helping us understanding the genome structure, gene order, and evolutionary lineages. Moreover, the addition of newly complete mitogenomes will contribute to our understanding of phylogenetic relationships. Regarding the relationships among families or subfamilies within Acrididae, a few hypotheses based on morphology have been presented (Flook and Rowell 1997b, 1998; Flook et al. 1999, 2000; Fenn et al. 2008; Ma et al. 2009; Sun et al. 2010; Zhao et al. 2010; Zhao et al. 2011; Li et al. 2012; Huang et al. 2013), but they were not always consistent with each other (Li et al. 2011; Chintauan-Marquier et al. 2014; Song et al. 2015). The lack of a consensus about phylogeny based only on morphology makes it especially critical to use DNA data from highly polymorphic genetic regions such as mitogenome sequences. The taxonomic and phylogenetic relationships of Melanoplinae have been studied by morphological and molecular data, but the systematics of the Podismini is still controversial (Litzenberger and Chapco 2001; Litzenberger and Chapco 2003; Shengquan et al. 2003; Chintauan-Marquier et al. 2011; Li et al. 2011; Chintauan-Marquier et al. 2014; Gen 2015; Grzywacz and Tatsuta 2017; Liu et al. 2017). The clades proposed by previous studies of the Podismini of Eurasian taxa do not fit the older morphological or cytological classifications but are in agreement with molecular studies (Litzenberger and Chapco 2001; Chintauan-Marquier et al. 2011; Chintauan-Marquier et al. 2014). However, some studies using different molecular markers may also give inconsistent results (Litzenberger and Chapco 2003; Grzywacz and Tatsuta 2017). The topological structure based on morphological data of Podismini was similar to that of the mtDNA and/or rDNA tree proposed previously (Gen 2015).

The genus *Sinopodisma* Chang, 1940 belongs to Melanoplinae, Acrididae, and Caelifera (Cigliano et al. 2019) based on the Orthoptera Species File (OSF). Approximately 42 species of *Sinopodisma* have been described and are mostly distributed in eastern Asia (Huang et al. 2013; Gen 2015). *Sinopodisma* grasshoppers are small in size, with degenerate wings; they mostly live in mountains 850 m above sea level (Wang et al. 2004). Based on morphological characters, *Sinopodisma* from China were divided into four groups (Shengquan et al. 2003). The results obtained from different data types failed to reach a consistent conclusion on the classification and evolutionary relationship of *Sinopodisma*, which needs further discussion. (Storozhenko 1993; Li et al. 2006; Li et al. 2011; Huang et al. 2013; Grzywacz and Tatsuta 2017; Liu et al. 2017; Chang et al. 2020).

In order to better understand the phylogenetic relationship of Melanoplinae, we obtained complete mitogenome sequences of *S. pieli*, *S. houshana*, *S. qinlingensis*, and *S. wulingshanensis* and compared them in detail. The new mitogenomes data not only helped us understand the characteristics of mitogenome of this group and the differences among different species, but also provided the basis for better exploring their evolutionary relationships. Combined with the new data and the existing data, we reconstructed the phylogeny of 53 Acrididae species based on a dataset of 37 complete mitochondrial genes, which may provide new angle for discussing the relationships within the Melanoplinae.

## Materials and methods

### Sample collection and DNA extraction

Information on the samples analysed in the present study is summarised in Suppl. material 1: Table S1. The samples were preserved in 100 % ethanol and stored at -20 °C freezer in Institute of Zoology of Shaanxi Normal University. Total genomic DNA was extracted from the muscle tissue of single individuals using the phenol/chloroform/isoamylalcohol method (Zhou et al. 2007), and then stored at -20 °C.

### DNA sequencing and annotations

High-Throughput Sequencing Technique was used to sequence *S. wulingshanensis*. We first fragmented DNA using an ultrasonic mechanical method. Then, we built DNA library and used Illumina HiSeq 2500 to sequence the whole genome, including the mitogenomes. The average read length was approximately 125 bp. The DNA library and sequencing were supported by the Biomarker Company (Kawahara and Breinholt 2014). At the same time, we obtained the mitogenome sequences of *S. pieli*, *S. houshana*, and *S. qinlingensis* by Sanger sequencing. We first synthesised Long-PCR(L-PCR) primers according to our own design, which divided the entire mitochondrial ring into six overlapping segments, each 3,000 bp to 4,500 bp long, covering the entire length of the whole mitochondrion, which is approximately 16,000 bp long (Suppl. material 1: Table S2) (Liu et al. 2006). Next, we used the mitochondrial universal primer sequences published by Simon (Simon et al. 1994; Simon et al. 2006) to perform sub-PCR, using the products of L-PCR as templates. The L-PCR was performed in a total volume of 25 μL including 11.25 μL ddH2O, 2.5 μL forward primer (10 μM), 2.5 μL reverse primer (10 μM), 1 μL template DNA (50 ng/μL), 2.5 μL dNTP Mixture (2.5 mM), 10 μL 10×LA PCR BufferII (Mg+Plus), and 0.25 μL TaKaRa LATaq DNA polymerase (5 μ/μL). The PCR reaction was under the following conditions: initial denaturation at 93 °C for 2 min → (92 °C for 10 sec, 52.5 °C for 30 sec, 68 °C for 8 min) × 20 cycles → (92 °C for 10 sec, 52.5 °C for 30 sec, 68 °C for 8 min + 20 sec) × 20 cycles → 72 °C for 7 min → decrease to 4 °C.

The sub-PCR was performed in a total volume of 40 μL including 14 μL of ddH2O, 2 μL of forward primer (10 μM), 2 μL of reverse primer (10 μM), 2 μL of template DNA (50 ng/μL), and 20 μL of 2×Taq PCRStar Mix. The sub-PCR was under the following conditions: initial denaturation at 96 °C for 2 min → (96 °C for 10 sec, 51.5 °C for 35 sec, 60 °C for 4 min) × 35 cycles → 72 °C for 7 min → decrease to 4 °C. Most sub-PCR products were directly sequenced by means of primer walking, and other fragments were cloned into the pGEM-T Easy vector (Promega, USA) prior to sequencing.

The Standen Package (Staden et al. 2000) was used for sequence assembly and annotation. Transfer RNAs were identified by tRNAscan-SE1.21 (Lowe and Eddy 1997), and the other genes were determined by comparison with other related mitogenome sequences. The sequences of PCGs were translated based on the invertebrate mtDNA genetic code. Sequence information analysis was performed using MEGA 6.0 (Tamura et al. 2013) and ClustalX2 (Larkin et al. 2007). With *S. wulingshanensis*, after sequencing the genomes of the two species, the raw reads were inserted in the CLC Genomics Workbench 9.0 (CLC Bio, Aarhus, Denmark) to trim reads and then saved as a fastq file. We used the mitogenome of *S. pieli* as the reference and assembled the mitogenome of *S. wulingshanensis* using Mira 4.0.2 and MITObim 1.7 (Hahn et al. 2013). We used Geneious Prime (Kearse et al. 2012) (Biomatters Ltd., Auckland, New Zealand) for mitogenome annotation. Tandem Repeats Finder (Benson 1999) online software (http://tandem.bu.edu/trf/trf.html) was used to predict repeat elements in A+T-rich region. The four mitogenome sequences are available at GenBank, accession numbers: KX857633, KX857634. KX857636, KX857637.

### Phylogenetic analyses

Fifty-three available insect mitogenomes were used the phylogenetic analyses of Acrididae. The mitogenomes of *Asiotmethis jubatus* (NC_025904), *Filchnerella beicki* (NC_024923) and *Humphaplotropis culaishanensis* (NC_023535) were downloaded and used as outgroup (Taxonomy of all species is based on Orthoptera Species File (Version 5.0/5.0) (Cigliano et al. 2019), Suppl. material 1: Table S3). We inferred phylogenies using all 37 gene sequences. Alignments of individual genes were concatenated using SequenceMatrix v1.7.8 (Gaurav et al. 2011). The concatenated matrix removing the stop codons was analysed by Bayesian Inference (BI) and Maximum Likelihood (ML) methods. Before phylogenetic analyses, the phylogenetic signals were assessed by MEGA6.0 (Tamura et al. 2013). For the ML analyses, PartitionFinder (Lanfear et al. 2012) was used to find the best-fit partitioning scheme for the 13 PCGs +2 rRNAs + 22 tRNAs data set. ModelFinder (Kalyaanamoorthy et al. 2017) was used to find best-fit partition models and phylogenetic tree were reconstructed by IQ-TREE 1.7 (Nguyen et al. 2015) with 1000 bootstrap replicates. For the BI analyses, we used the best-fit partitioning scheme and partition-specific models recommended by PartitionFinder (Lanfear et al. 2012) and analysed using MrBayes 3.2.6 (Ronquist and Huelsenbeck 2003) with the MCMC analysis run for 2,000,000 generations and sampling every 1000 trees. After discarding the first 25% samples as burn-in, posterior probabilities (PP) were calculated in a consensus tree.

## Results and discussion

### Mitogenome organisation

**Figure 1.**
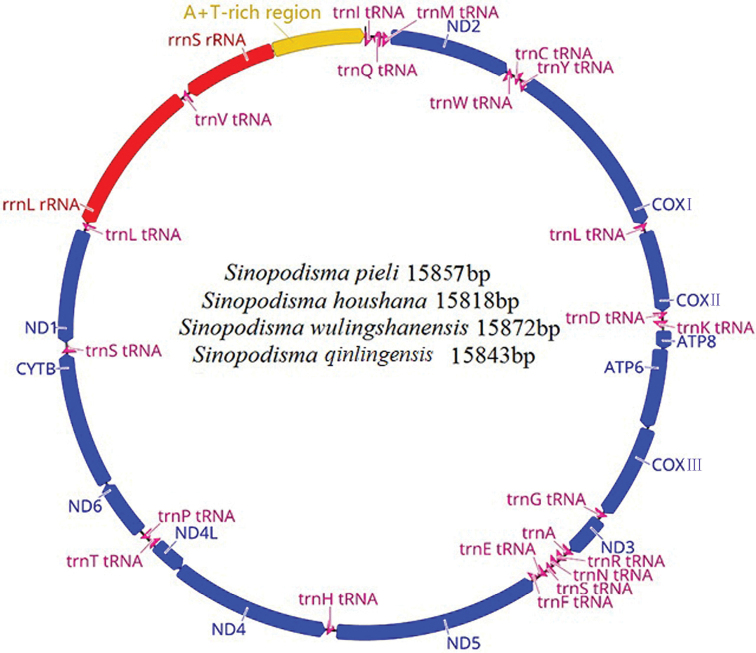
Circular map of the mitogenome from four species.

The complete mitogenomes of *S. pieli*, *S. houshana*, *S. wulingshanensis* and *S. qinlingensis* are 15,857 bp, 15,818 bp, 15,872 bp and 15,843 bp in size, respectively (Figure 1, Suppl. material 1: Tables S4, S5). All mitogenomes contain a conserved set of 37 genes, including 13 PCGs (ATP6, ATP8, COXI, COXII, COXIII, CYTB, ND1, ND2, ND3, ND4, ND5, ND6, and ND4L), rRNAs (rrnL and rrnS), 22 tRNAs and a large non-coding region called the A+T-rich region. All four species have the arrangement order translocation of trnK and trnD (Figure 1). The gene overlapping between the four mitogenomes ranges from 1 to 8 bp in size. The longest overlapping region in the four mitogenomes, with a length of 33 bp, is located between trnY and COXI, except in *S. pieli*, where it is located between trnY-COXI and trnW-trnC. Intergenic regions (IGRs) of the four species range from 1 to 16 bp in size, with the longest IGRs located between trnSUCN and ND1. The A+T content, AT skew and GC skew exhibit similar characteristics in the four species (Table 1).

### Nucleotide compositions

Generally, A % > T % and C % > G % are common characteristics in all insects (Li et al. 2012). The nucleotide compositions of the four species’ mitogenomes are significantly biased towards A and T (A+T > 76 %, see Table 1). All four mitogenomes (measured on the major strand) favour A-skew and C-skew. The A+T content (as measured on the major strand) among the four sequenced mitogenomes is 76.8 % (*S. qinlingensis*), followed by 76.5 % (*S. wulingshanensis*), 76.4 % (*S. pieli*) and 76.4 % (*S. houshana*) (Suppl. material 2: Figure S1). The lowest A+T content of orthopteran mitogenomes is 63.3 % in *Gampsocleis gratiosa*. Compared to the mitogenomes of other insects, they have very high contents of AT bases. In addition to the important effect of the GC content on the stability of double-stranded DNA, some researchers surmise that the C→T mutation in the mitogenome of insects leads to the high AT contents of the mitochondrial genes (Consortium 2006). Research on vertebrate genomes suggests that biased codon usage drives the evolution of tRNA anticodons, but those patterns cannot be generalised to invertebrate mitogenomes due to the differences between vertebrate and invertebrate mtDNA; therefore, this assumption is not final conclusion (Xia 2005). Furthermore, the GC content of the mitogenome does not reflect taxonomic characteristics or evolutionary relationships.

**Table 1.** Nucleotide compositions of *S. pieli*, *S. houshana*, *S. wulingshanensis*, and *S. qinlingensis*.

Feature	AT%				AT-skew				GC-skew			
	*S. q.*	*S. w.*	*S. p.*	*S. h.*	*S. q.*	*S. w.*	*S. p.*	*S. h.*	*S. q.*	*S. w.*	*S. p.*	*S. h.*
Whole genome (J-strand)	76.8	76.5	76.4	76.4	0.121	0.126	0.121	0.126	-0.122	-0.126	-0.126	-0.129
Protein-coding genes*	76.1	75.7	75.8	75.6	-0.14	-0.141	-0.143	-0.14	0.021	-0.001	0.004	0.019
First codon position	68.8	68.4	66.3	68	-0.039	-0.042	-0.296	-0.046	0.24	0.214	-0.06	0.241
Second codon position	65.9	65.6	86.8	65.5	-0.403	-0.407	-0.142	-0.404	-0.171	-0.169	-0.202	-0.169
Third codon position	93.8	93.2	74.4	93.3	-0.029	-0.026	-0.006	-0.023	-0.026	-0.154	0.194	-0.073
22 tRNAs	74	74.5	73.9	74.2	0.011	0.017	0.016	0.005	0.112	0.103	0.1	0.1
2 rRNA	76.7	76.4	75.7	76.3	-0.141	-0.157	-0.134	-0.154	0.21	0.215	0.208	0.23
*rrnL*	77.5	77.6	77.7	77.5	-0.171	-0.183	-0.149	-0.172	0.22	0.218	0.213	0.218
*rrnS*	75.4	74.3	72.5	74.2	-0.092	-0.111	-0.108	-0.124	0.194	0.21	0.202	0.243
A+T-rich region	86.3	86.6	86.5	86.5	0.146	0.16	0.152	0.154	-0.165	-0.167	-0.147	-0.141

Notes: * Stop codons were excluded. AT%= [A+T]/[A+T+G+C], ATskew=[A−T]/[A+T],GC-skew=[G−C]/[G+C].

### Protein-coding genes

A total of nine PCGs (ND2, COXI, COXII, ATP8, ATP6, COXIII, ND3, ND6 and CYTB) are located on the J-strand, while the others (ND5, ND4, ND4L and ND1) are located on the N-strand. The 13 PCGs start with typical ATN codons in all four species, except COXI in *S. qinlingensis*, which begins with ACC. Many nonstandard initiation codons have previously been reported (Nardi et al. 2003; Kim et al. 2005; Samuels et al. 2005; Fenn et al. 2007; Ma et al. 2009; Liu and Huang 2010; Sheffield et al. 2010; Yang and Huang 2011; Ye et al. 2012; Zhang et al. 2013; Kim et al. 2014), including ATTTAA (Nardi et al. 2003), ATTA (Ye et al. 2012), TTAA and ATTA (Fenn et al. 2007), CCG (Zhang et al. 2013), CGA , AAA (Liu and Huang 2010), GTG (Samuels et al. 2005). The 13 PCGs of the four species all terminate with the conventional stop codons TAG or TAA.

The A+T content of the 13 PCGs, excluding stop codons, is observed to be 76.1%, 75.7%, 75.8% and 75.6% in *S. qinlingensis*, *S. wulingshanensis*, *S. pieli* and *S. houshana*, respectively (Table 1). The highest A+T content is found in the third codon position (93.8% in *S. qinlingensis*, 93.2% in *S. wulingshanensis* and 93.3% in *S. houshana*), but in *S. pieli*, the highest A+T content is in the second position (86.8%). The most obvious T-skew is recovered in the second codon position, except *S. pieli*. Additionally, the three codon positions show different GC-skews. In all four species other than *S. pieli*, the first codon position exhibits G-skew, and the other two codon positions exhibit C-skew. In *S. pieli*PCGs, the first and second codon positions exhibit C-skew, and the third codon position exhibits G-skew.

**Figure 2.**
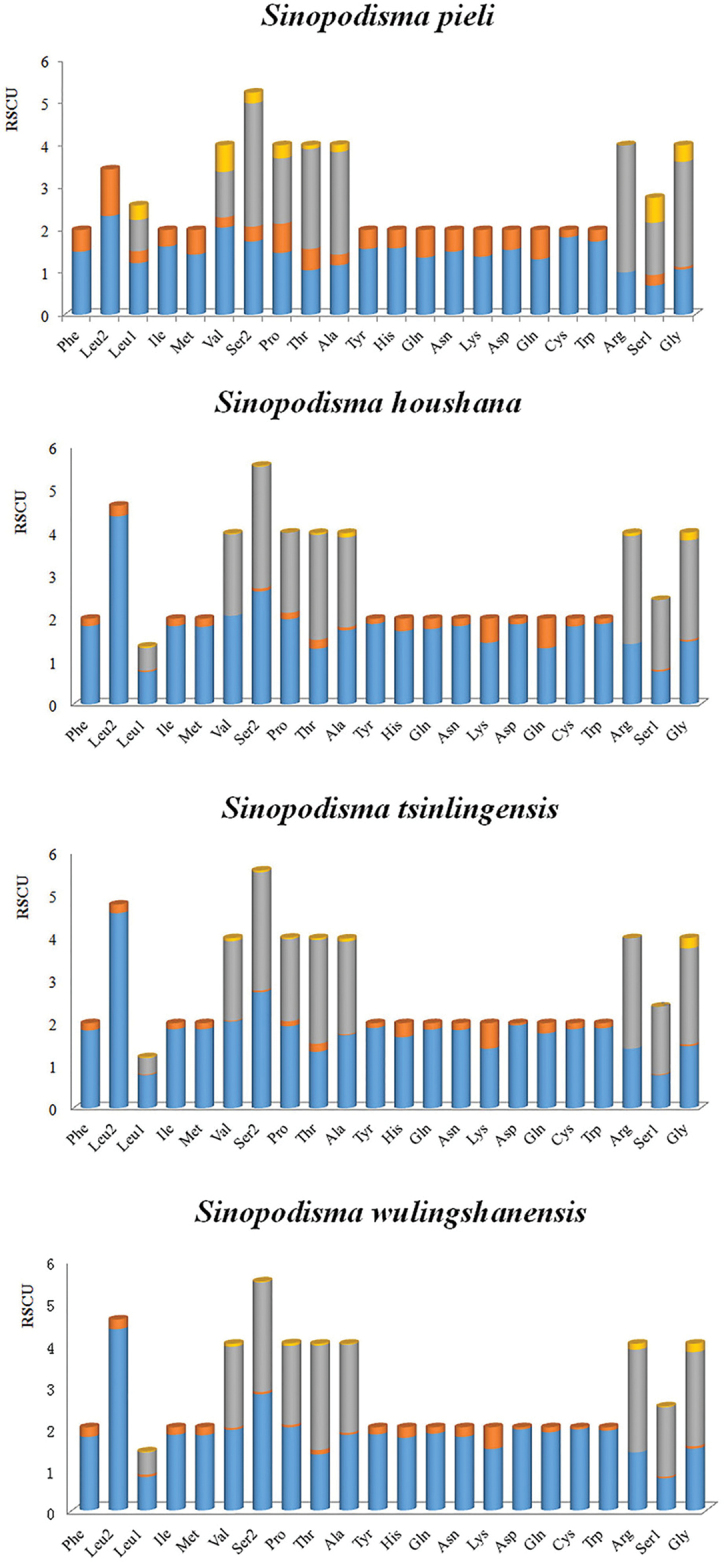
The relative synonymous codon frequencies from the four species.

Codon usage bias (codon bias) is a phenomenon in which specific codons are used more frequently than other synonymous codons by certain organisms during the translation of genes to proteins. With rapid progress in whole-genome sequencing, analysis of codon usage bias at the genome level, rather than for a single gene or a set of genes, has gained attention. Genome-wide investigations on the variations in codon use and codon context bias are important for understanding the functional evolution of genomes within and between species (Lu et al. 2002; Fenn et al. 2007; Sun et al. 2017; Xu et al. 2019). Relative synonymous codon usage (RSCU) analysis indicated that in katydids, codons including A or T at the third position are always overused compared with other synonymous codons. The biased usage of AT nucleotides is also reflected in the form of codon usage, with RSCU values negatively correlating with the C and G contents in codons. The relatively synonymous codon frequencies of the four species’ PCGs were summarised in Figure 2. Among the 64 available codons, the most frequently used codons are TTA (Leu), ATT (Ile), TTT (Phe), and ATA (Met), which are composed entirely of AT nucleotides. The codon CGC (Arg) is not used by the PCGs of *S. pieli*, *S. qinlingensis*, *S. wulingshanensis* or *S. houshana* mitogenomes. The codon CGG (Arg) is not found in *S. pieli* or *S. qinlingensis* mitogenomes. The codon AGG (Ser) is not exist in *S. qinlingensis* or *S. houshana* mitogenomes. The codons CCG (Pro) and GCU (Val) do not appear in the *S. houshana* mitogenome. To adapt to different living habits or types of resistance, different species require different functions of proteins, inevitably resulting in the preferential use of amino acids. In order from most to least frequent, the four mostly used amino acids in *S. houshana*, *S. wulingshanensis* and *S. qinlingensis* are Leu, Ile, Ser, and Phe; by contrast, the four mostly used amino acids in *S. pieli* are Leu, Phe, Tyr and Ile (Suppl. material 2: Figure S2).

### Transfer RNA genes

A total of 14 tRNAs (trnI, trnM, trnW, trnL^UUR^, trnD, trnK, trnG, trnA, trnR, trnN, trnS^AGN^, trnE, trnT, trnS^UCN^) are located on the J-strand, while the remaining tRNAs (trnQ, trnC, trnY, trnF, trnH, trnP, trnL^CUN^, trnV) are located on the N-strand. Moreover, 21 of the 22 tRNAs are well folded into a clover-leaf-like secondary structure, except trnS^AGN^, which lacks the DHU stem in all four species (Suppl. material 2: Figure S3). This phenomenon is considered to be a typical feature of metazoan mitogenomes (Wolstenholme 1992). In *S. houshana*, trnP is only 58 bp, and its variable (V) loop and TΨC arm are incomplete. Due to the stereoscopic limitations, it could not form a stable hydrogen bond in the TΨC arm. Therefore, the stereoscopic structure of this tRNA apparently only contains three arms, and the variable (V) loop and TΨC arm merged into an armband structure with 10 bp using a weaker hydrogen bond (Suppl. material 2: Figure S3). Additionally, trnK and trnD are translocated with each other in four mitogenomes. The tRNA translocation trnD-trnK seems to be a synapomorphy of the caeliferan group Acridomorpha (Song et al. 2015).

The lengths of the 22 tRNAs in the four species range from 64 to 71 bp in *S. pieli*, *S. qinlingensis* and *S. wulingshanensis*, and from 58 to 71 bp in *S. houshana*. According to the secondary structures and sequence alignments, the most conserved tRNAs in the four mitogenomes is trnF (Suppl. material 2: Figure S3), with the same nucleotide, trnG with one nucleotide insertion, and trnL^CUN^, with one nucleotide substitution.

In the remaining tRNAs, nucleotide substitutions are mainly restricted to loops, with obvious insertion-deletion polymorphisms. In *S. pieli*, there are 27 non-canonical base pairs, consisting of 17 G-U pairs and 1 A-A, 2 A-G, 1 A-C, 1 U-C and 5 U-U mismatches. In *S. qinlingensis*, there are 21 non-canonical base pairs, consisting of 17 G-U pairs and 1 A-A, 1 A-G and 2 U-U mismatches. In *S. wulingshanensis*, there are 22 non-canonical base pairs, consisting of 18 G-U pairs and 1 A-A, 1 A-G and 2 U-U mismatches. In *S. houshana*, there are 27 non-canonical base pairs, consisting of 19 G-U pairs and 1 A-A, 2 A-G, 2 A-C, 1 U-C and 2 U-U mismatches. The possession of aberrant mismatches, loops, or extremely short arms for tRNA is common in metazoan mitogenomes (Wolstenholme 1992). Although it remains unknown whether the aberrant tRNAs lose their respective functions, that could be corrected by post-transcriptional RNA editing processes (Masta and Boore 2004).

### Ribosomal RNA genes

Similar to other insect mitogenomes, rrnL is located between trnL^CUN^ and trnV, and rrnS is located between trnV and the A+T-rich region. The lengths of rrnL are 1,343 bp, 1,313 bp, 1,369 bp and 1,313 bp in the *S. pieli*, *S. houshana*, *S. wulingshanensis* and *S. qinlingensis* mitogenomes, respectively, and the lengths of rrnS are 792 bp, 797 bp, 797 bp, and 797 bp, respectively. In the other orthopteran mitogenomes in GenBank, the lengths of rrnL range from 1,236 bp (*Gryllotalpa pluvialis*, NC_011302) to 1,371 bp (*Pseudoxya diminuta*, NC_025765), and the lengths of rrnS range from 461 bp (*Ceracris kiangsu*, NC_019994) to 882 bp (*Ruspolia dubia*, NC_009876). Therefore, the lengths of rrnL and rrnS from these four species are within the normal range. The A+T content ranges from 72.5% to 77.7% in the rRNA genes, and both rRNA genes exhibit T-skew and G-skew (Table 1).

### A+T-rich region

**Figure 3.**
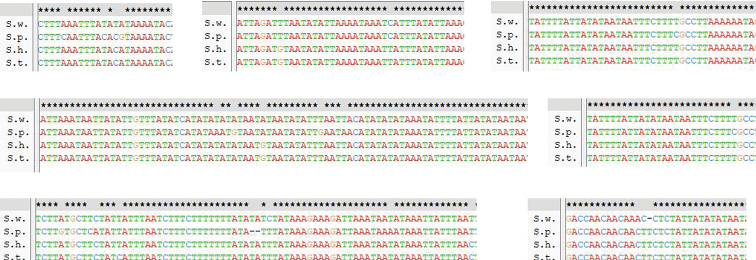
The long polythymine stretch and conserved sequence blocks in the A+T rich regions from four species. Note: The long polythymine stretch. T-stretch sequence was labelled with box, located in the majority strand. Within each block, nucleotides identical in the two sequences are bottom-marked with asterisks.

The A+T-rich region is the major noncoding region in insect mitogenome, which is located in the conserved position between the rrnS and trnI genes and has an A+T content of 86.5% in *S. pieli* and *S. houshana*, 86.6% in *S. wulingshanensis* and 86.3% in *S. qinlingensis*. In addition, the A+T-rich regions of all four mitogenomes favour A-skew and C-skew (Suppl. material 1: Table S5). The A+T-rich region is considered to be involved in the regulation of mtDNA transcription and replication, and there is a long polythymine stretch (T-stretch) adjacent to the trnI gene. A similar T-stretch has been found in some other insects, including *A. zacharjini* (17 bp), *F. helanshanensis* (14 bp) and *P. rubimarginis* (14 bp) (Zhang et al. 2013). The T-stretches on the major strand are also found in the A+T-rich region in our four sequenced mitogenomes, with a length of 11 bp but with two Cs inserted. However, they are not adjacent to the trnI locus but are instead inside the stem-loop sequence in the major strand (Figure 3).

Some tandem repetition and conserved structural elements have been observed in the insect A+T-rich region. Comparison of our four species with *Schistocerca gregaria* and *Oxya chinensis* revealed some conserved blocks. Indeed, these A+T-rich regions have eight conserved blocks (Figure 3). Blocks E1 and E2 can form a highly conserved stem-loop secondary structure in which the stem consists of 12 base pairs (Suppl. material 2: Figure S4). However, we do not find the motif “TATA” at the 5' end or the motif “G(A)nT” at the 3' end. The A+T-rich region of mitogenome may have evolved to have some alternative flanking sequence forms, if it had been present for a functional role (Yang et al. 2011).

**Figure 4.**
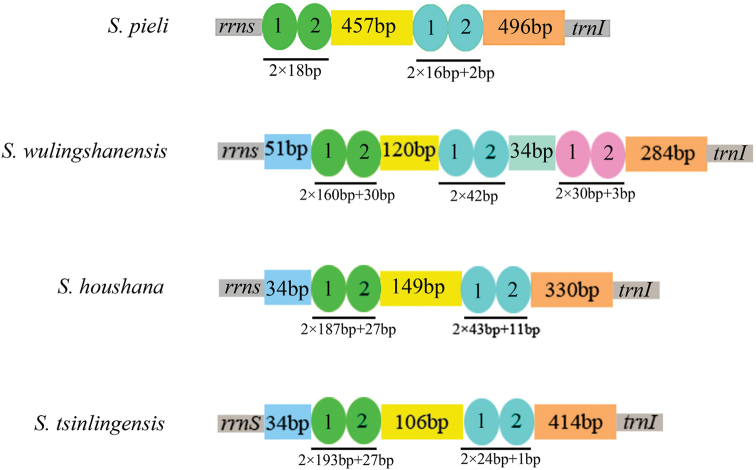
The main repeat elements in A+T-rich regions from four species.

The presence of a variable number of tandem repeat units may be useful for inferring the genetic structures of populations among closely related taxa and individuals of the same species (Mancini et al. 2008). Two repeated elements are found in the A+T-rich region of the *S. pieli* mitogenome. The A+T-rich regions of *S. houshana* and *S. qinlingensis* have six repeated elements, whereas the A+T-rich region in *S. wulingshanensis* contains three repeated elements (Suppl. material 1: Table S6). Tandem repeats may play various regulatory and evolutionary roles (Armour et al. 1996; Benson 1999); the main tandem repeats of the four species are shown in Figure 4.

### Phylogenetic relationships

**Figure 5.**
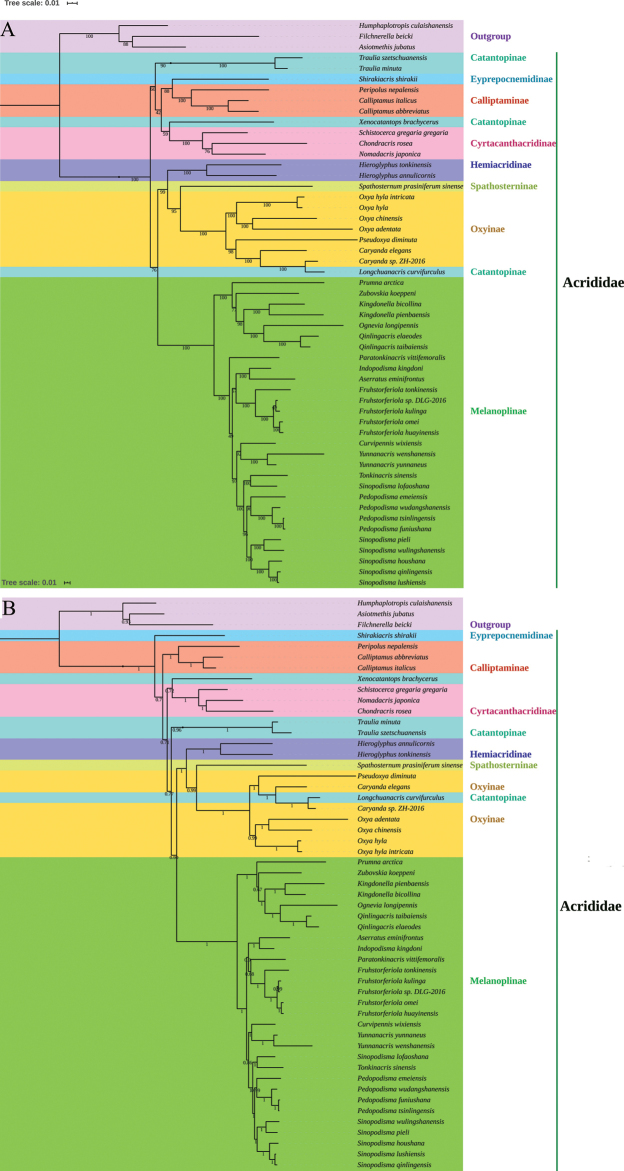
The phylogenetic tree based on 37 mitochondrial genes. (**A**) ML tree; (**B**) BI tree.

The dataset of all 37 mitochondrial genes was used to perform phylogenetic analyses based on 53 Acrididae mitogenome sequences, including the four newly generated sequences, 49 other Acrididae sequences from GenBank and three outgroup sequences (Suppl. material 1: Table S3). The partition of dataset and their optimal models were shown in Suppl. material 1: Table S7. There were some differences in the topologies of ML and BI trees (Figure 5). The relationships between Cyrtacanthacridinae, Eyprepocnemidinae, Calliptaminae, and Catantopinae and the relationships between the four subfamilies and other subfamilies were quite different in the two trees. Based on the tree topologies, Melanoplinae species clustered together, supporting the monophyly of this subfamily. Among the other subfamilies, the monophyly of Hemiacridinae, Cyrtacanthacridinae, and Calliptaminae could be supported, but the monophyly of the other four subfamilies (Catantopinae, Oxyinae, Spathosterninae, and Eyprepocnemidinae) could not be tested (Figure 5).

In Melanoplinae, the topological relationships between the genera were the same in the two trees and all species belong to the tribe Podismini. The phylogenetic results in this study supported the monophyly of Podismini. The same inference could be found in other phylogenies based on mitogenome data (Zhang and Lin 2016; Zhang et al. 2017), but the topology based on single gene did not support the monophyly of Podismini (Chintauan-Marquier et al. 2014; Grzywacz and Tatsuta 2017). Our topologies showed that *Sinopodisma* clustered two groups: (1) group 1: (((*S. lushiensis* + *S. qinlingensis*) + *S. houshana*) + (*S. pieli* + *S. wulingshanensis*)) and (2) group 2: (*S. lushiensis*+ *Tonkinacris sinensis*). So *Sinopodisma* do not cluster a monophyletic taxon, which was consistent with the results of Liu et al. (2017). But it was not agreement with other research results (Huang et al. 2013; Grzywacz and Tatsuta 2017). The present phylogenetic trees placed *Sinopodisma* as an apical node sister to *Pedopodisma*. *Sinopodisma*, and *Pedopodisma* are similar in morphology and distinguished only by slight differences (Huang et al. 2013; Cigliano et al. 2019). *Pedopodisma*, a genus endemic to China, was synonymized by Storozhenko (1993), which caused confusion on the classification of genus *Sinopodisma*. Storozhenko’s treatment has not been accepted by Chinese acridologists (Li et al. 2006) because tegmina are completely absent in Pedopodisma but distinct though reduced in Sinobodisma (Huang et al. 2013). Clearly, we only obtained six mitogenomes of *Sinopodisma*, and four of *Pedopodisma*, so additional sampling of the taxa *Sinopodisma* and *Pedopodisma* is needed to obtain sufficient mitogenome data to clarify the monophyly between *Sinopodisma* and the phylogenetic relationship of *Sinopodisma* and *Pedopodisma*.

## Conclusions

The complete mitochondrial genomes of *Sinopodisma pieli*, *S. houshana*, *S. qinlingensis*, and *S. wulingshanensis* were obtained. The mitogenomes of four species have typical genome organisation and gene arrangement order, compared to other caeliferan mitogenomes. We focused on comparative analyses of four *Sinopodisma* mitogenomes to find the characteristics of base composition, overlapping and intergenic regions, and tRNA secondary structures. All 13 PCGs have typical starting ATN codons, except for COXI in *S. qinlingensis*, which start with ACC. A+T contents in four mitogenomes are high and we found several repeated elements in the A+T-rich region of the four species. Moreover, 53 mitogenome data were used to build the phylogenetic relationship. The phylogenetic tree supported the monophyly of Melanoplinae, but do not support the monophyly of *Sinopodisma*.

## Supplementary materials

Supplementary material 1 Tables S1–S7 Data type table excel Explanation note **Table S1.** Information on the samples analysed in the present study. **Table S2.** List of L-PCR primers used in this study. **Table S3.** Taxonomic information and GenBank accession numbers for the 53 taxa used for the phylogenetic analysis in this study. **Table S4.** Annotation and gene organisation of four mitochondrial genomes. **Table S5.** Base composition and length features of the four mitochondrial genomes used in this study. **Table S6.** The repeat elements in A+T-rich regions of four species. **Table S7.** Subset partition and its optimal model of datasets. https://binary.pensoft.net/file/453682This dataset is made available under the Open Database License (http://opendatacommons.org/licenses/odbl/1.0/). The Open Database License (ODbL) is a license agreement intended to allow users to freely share, modify, and use this Dataset while maintaining this same freedom for others, provided that the original source and author(s) are credited.Qiu Zhongying, Chang Huihui, Yuan Hao, Huang Yuan, Lu Huimeng, Li Xia, Gou Xingchun

Supplementary material 2 Figures S1–S4 Data type images Explanation note **Figure S1.** The A+T content of mitogenomes in orthopteran species. **Figure S2.** The amino acid composition from *S. pieli*, *S. houshana*, *S. wulingshanensis*, and *S. qinlingensis*. **Figure S3.** Inferred secondary structure of tRNA families in *S. pieli*, *S. houshana*, *S. wulingshanensis* and *S. qinlingensis* mitochondrial genomes. Note: The nucleotide substitution pattern for each tRNA family is modeled using as the reference the structure determined for *S. pieli*. Because of the special secondary structure of trnP in *S. houshana*, the trnP in Figure S3 is presented separately. In nucleotide substitutions and insertions, we used different colours to represent different species (one or several). The colour of some substitutions and insertions only represent some species, which means base deletions in the location of other species. **Figure S4.** Putative stem-loop structures found in A+T-rich region from four species. https://binary.pensoft.net/file/453683This dataset is made available under the Open Database License (http://opendatacommons.org/licenses/odbl/1.0/). The Open Database License (ODbL) is a license agreement intended to allow users to freely share, modify, and use this Dataset while maintaining this same freedom for others, provided that the original source and author(s) are credited.Qiu Zhongying, Chang Huihui, Yuan Hao, Huang Yuan, Lu Huimeng, Li Xia, Gou Xingchun
